# Incidental Detection of Hepatocellular Carcinoma by Echocardiographic Imaging During Acute Coronary Syndrome Evaluation

**DOI:** 10.7759/cureus.112020

**Published:** 2026-07-03

**Authors:** Abdulrahman Alharbi, Munthir Alahmed, Waleed A Elsaadani, Hussein AlMejbel

**Affiliations:** 1 Internal Medicine, Security Forces Hospital Makkah, Makkah, SAU; 2 Cardiology, Security Forces Hospital Makkah, Makkah, SAU

**Keywords:** hepatocellular carcinoma, incidental finding, inferior vena cava mass, subcostal view in echocardiography, unstable angina

## Abstract

Hepatocellular carcinoma is a primary liver malignancy that may invade vascular structures, including the inferior vena cava, in advanced stages. Detection during cardiac imaging is uncommon. A 73-year-old man with hypertension and type 2 diabetes mellitus presented with chest pain suggestive of acute coronary syndrome. Initial electrocardiographic assessment and cardiac biomarkers showed no evidence of myocardial injury. During transthoracic echocardiographic evaluation, an unexpected mass was identified within the inferior vena cava on the subcostal view. Subsequent contrast-enhanced computed tomography demonstrated a large right hepatic lobe mass with characteristic arterial enhancement and portal venous/delayed-phase washout, along with inferior vena cava invasion. Histopathological examination confirmed hepatocellular carcinoma. This case illustrates the importance of a systematic echocardiographic approach, as incidental extracardiac findings may reveal significant underlying disease and alter clinical management.

## Introduction

Hepatocellular carcinoma represents the dominant form of primary liver cancer and continues to contribute substantially to global cancer mortality [1]. Its occurrence is strongly linked to chronic liver disease, particularly in individuals with long-standing viral hepatitis infections or cirrhosis of varying etiologies [2]. Although surveillance programs have improved early detection in high-risk populations, a proportion of patients still present at later stages due to subtle or nonspecific clinical manifestations such as fatigue, abdominal discomfort, or weight loss [3,4].

Tumor invasion into vascular structures is a key indicator of aggressive disease behavior in hepatocellular carcinoma. While involvement of the portal vein is most frequently encountered, extension into the hepatic veins and inferior vena cava is less common but carries more serious clinical implications [5]. Such vascular spread is typically associated with advanced-stage disease and may predispose patients to complications, including systemic dissemination and features resembling hepatic venous outflow obstruction [6].

Unexpected identification of hepatocellular carcinoma during investigations for unrelated conditions is rare. Even more unusual is its detection during cardiac imaging studies. In this report, we describe a patient undergoing evaluation for suspected acute coronary syndrome in whom transthoracic echocardiography revealed an inferior vena cava mass. Further diagnostic work-up confirmed an underlying hepatic malignancy with vascular invasion. Although the subcostal view during transthoracic echocardiography is routinely used to evaluate cardiac anatomy, detect pericardial effusion, and assess the inferior vena cava for estimation of right atrial pressure and volume status, it also visualizes adjacent abdominal structures and may reveal clinically important extracardiac findings, including pleural effusions, ascites, liver lesions, and inferior vena cava thrombi or tumor extension [7,8]. The incidental detection of hepatocellular carcinoma during evaluation for suspected acute coronary syndrome is exceptionally uncommon and highlights the importance of systematically assessing all structures within the imaging field. This case underscores the broader diagnostic value of echocardiography beyond cardiac assessment, particularly when subcostal views are carefully evaluated.

## Case presentation

A 73-year-old male with a history of hypertension and type 2 diabetes mellitus presented to the emergency department with typical retrosternal chest pain of one day's duration, radiating to the neck and left shoulder, and associated with diaphoresis. He denied dyspnea, palpitations, or syncope.

On physical examination, he was conscious and alert. Vital signs were as follows: blood pressure 130/70 mmHg, heart rate 90 bpm, respiratory rate 20 breaths/min, and oxygen saturation 96% on room air. Chest auscultation revealed equal bilateral air entry with normal vesicular breath sounds. Cardiac examination demonstrated normal S1 and S2 without additional sounds. The abdomen was soft and nontender, with no hepatosplenomegaly. The remainder of the systemic examination was unremarkable.

Initial investigations revealed a normal sinus rhythm without ischemic changes on electrocardiography (Figure 1). 

**Figure 1:**
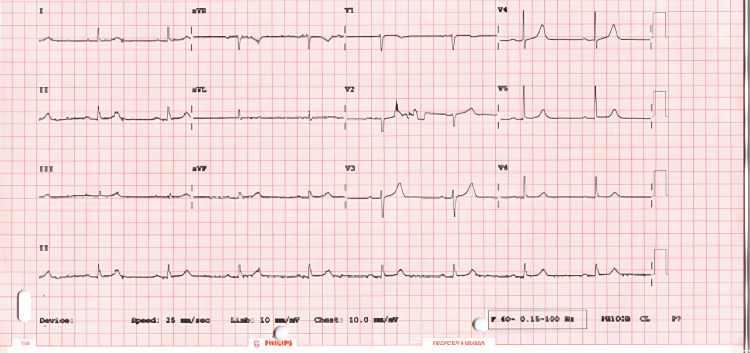
ECG showing normal sinus rhythm with no ST-T segment changes.

Laboratory findings were largely within normal limits, except for elevated liver enzymes, as summarized in Table 1. 

**Table 1:** Laboratory results.

Test Item	Reference Range	Unit	Result
Troponin I (high sensitivity)	0-0.026	ng/mL	<0.01
BNP	0-160	pg/mL	100
Hemoglobin	13.5-17.2	g/dL	12.6
Platelets	150,000-450,000	/μL	170,000
WBC	4,000-11,000	/μL	4,500
Creatinine	63.6-110.5	µmol/L	89
Aspartate aminotransferase (AST)	5-34	U/L	161
Alanine aminotransferase (ALT)	0-55	U/L	56
Total bilirubin	3.4-20.5	µmol/L	10.3
Alpha-fetoprotein (AFP)	0.89-8.78	ng/mL	83

The patient was admitted to the coronary care unit with a working diagnosis of unstable angina, and standard acute coronary syndrome therapy was initiated. After stabilization and resolution of chest pain, transthoracic echocardiography was performed to assess for wall motion abnormalities.

The study demonstrated preserved left ventricular function with no regional wall motion abnormalities. However, the subcostal view revealed an echogenic mass within the inferior vena cava (IVC), raising suspicion for thrombus or tumor (Figures 2, 3). 

**Figure 2:**
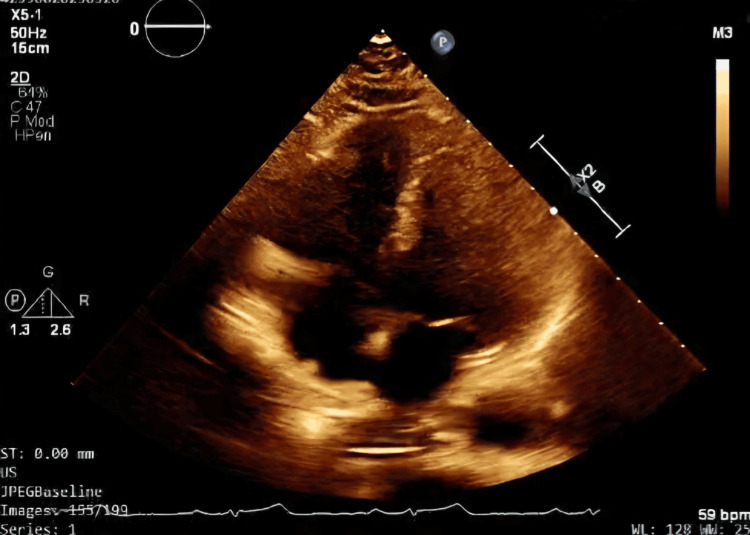
Transthoracic echocardiography. Apical four-chamber view demonstrating preserved left ventricular function, no regional wall motion abnormalities, and no masses within the right heart.

**Figure 3:**
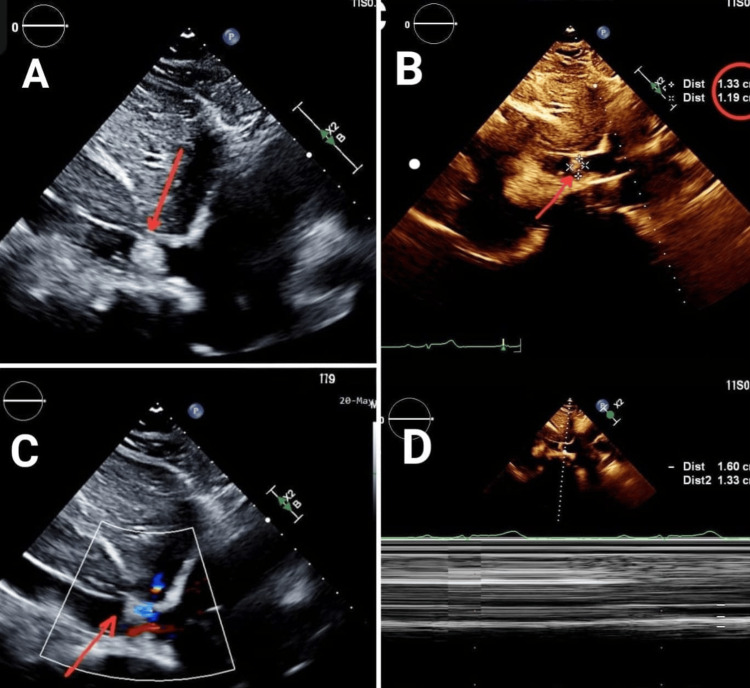
Transthoracic echocardiography. (A-C) Subcostal views showing an inferior vena cava (IVC) mass measuring 1.33 × 1.19 cm, suspicious for thrombus or tumor. Red arrows indicate the IVC mass, and the circle represents its diameter. (D) Subcostal view with M-mode imaging of the IVC demonstrating a normal IVC diameter of 1.6 cm without dilatation.

As part of a malignancy workup, contrast-enhanced computed tomography (CT) was performed. The abdominal phase revealed a large exophytic hepatic mass (~8.5 × 7 cm) in the right lobe, demonstrating heterogeneous arterial enhancement with portal venous and delayed-phase washout, consistent with hepatocellular carcinoma. Additional smaller enhancing lesions were identified in segment V (1.3 cm) and segment VI (1.2 cm). Tumor invasion into the inferior vena cava and occlusion of the right hepatic vein were also noted. No thoracic or pelvic metastases or lymphadenopathy were detected (Figure 4). 

**Figure 4:**
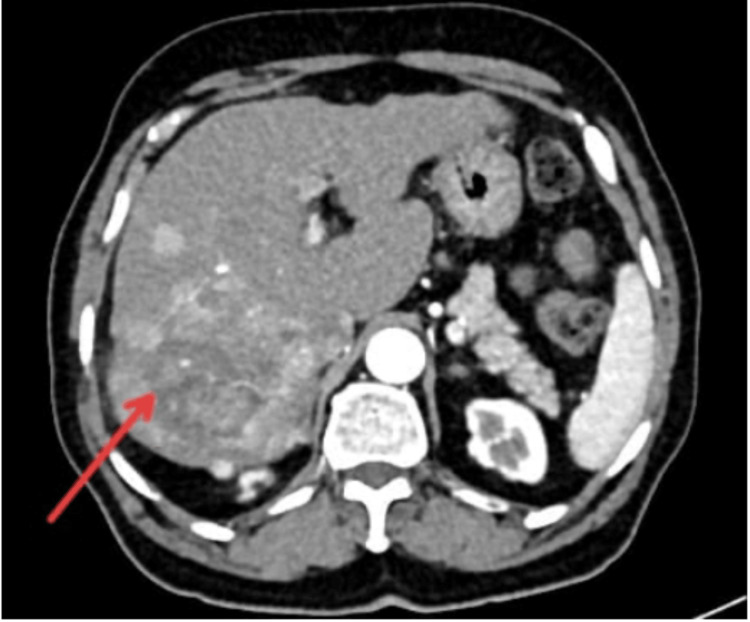
Contrast-enhanced CT of the abdomen demonstrating a large heterogeneous exophytic hepatic mass (red arrow) measuring 8.5 × 7 cm in the right hepatic lobe. The lesion shows heterogeneous arterial phase enhancement with portal venous and delayed phase washout, consistent with hepatocellular carcinoma. The tumor extends into the inferior vena cava at the suprahepatic level without extension into the right atrium.

Alpha-fetoprotein was elevated (Table 1). Ultrasound-guided core needle biopsy confirmed hepatocellular carcinoma, showing malignant epithelial cells arranged in trabecular and pseudoglandular patterns, with nuclear pleomorphism, prominent nucleoli, and bile pigment. Reticulin staining demonstrated architectural disruption, while CD34 immunostaining confirmed diffuse sinusoidal capillarization.

Coronary CT angiography revealed mild non-obstructive coronary artery disease, supporting conservative management of acute coronary syndrome (Figure 5). The patient was subsequently referred to oncology and hepatology for multidisciplinary management because this incidental finding changed the management plan according to the tumor stage and the risk of bleeding. 

**Figure 5:**
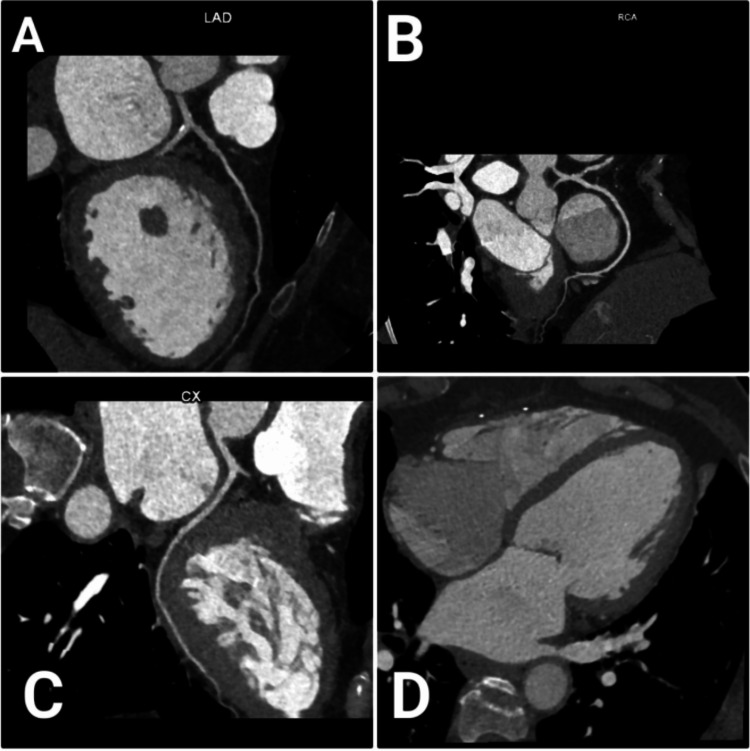
Coronary computed tomography angiography (CCTA). Images (A-D) reveal minimal stenosis of the left main coronary artery (LM), proximal and mid left anterior descending artery (LAD), right coronary artery (RCA), and proximal left circumflex artery (LCX).

## Discussion

The presence of vascular invasion in hepatocellular carcinoma signifies advanced disease and is associated with limited therapeutic options and reduced survival. In addition, the extent of vascular involvement may influence disease burden and subsequent treatment planning [6]. While portal vein involvement is more commonly observed, extension into the inferior vena cava (IVC) represents a more aggressive pattern of tumor progression and carries a higher risk of serious complications, including embolic events and potential cardiac involvement. Recognition of this pattern is essential, as it directly impacts staging, prognosis, and therapeutic decision-making [7].

Transthoracic echocardiography is primarily utilized for cardiac evaluation; however, its diagnostic utility extends beyond the heart. Incidental extracardiac findings, although relatively uncommon, can provide clinically significant information. These may include pleural effusions, intra-abdominal fluid collections, or abnormalities involving the liver and major vascular structures. Previous studies have shown that such findings occur in a small but meaningful proportion of echocardiographic examinations and may alter subsequent clinical management [8].

The subcostal acoustic window, in particular, offers a valuable perspective for visualization of the inferior vena cava and adjacent hepatic anatomy. Although it is routinely used for estimating right atrial pressure and assessing volume status, it may also reveal unexpected abnormalities such as intravascular masses or hepatic lesions [9]. Careful and systematic evaluation of this view is therefore crucial, especially when image quality is adequate.

In the present case, the incidental detection of an IVC mass during echocardiographic assessment prompted further investigation, ultimately leading to the diagnosis of hepatocellular carcinoma with vascular invasion. Similar cases have been reported in the literature, highlighting the role of echocardiography in identifying noncardiac malignancies when tumors extend into vascular structures within the imaging field [10,11]. This underscores the importance of maintaining a comprehensive and methodical approach during echocardiographic examinations, as clinically significant findings may lie outside the primary focus of interest.

## Conclusions

This case illustrates how an apparently straightforward cardiac presentation can uncover a serious underlying noncardiac condition. Evaluation for suspected acute coronary syndrome led to the unexpected identification of an inferior vena cava mass, which was subsequently diagnosed as hepatocellular carcinoma with vascular invasion.

It highlights the importance of maintaining a broad clinical perspective and thoroughly assessing all echocardiographic views, particularly the subcostal window. Although incidental findings are uncommon, they can significantly alter the diagnostic pathway and overall patient management. Early recognition of such findings facilitates timely referral and multidisciplinary care, which are especially critical in advanced hepatocellular carcinoma, where prognosis is closely linked to early detection and appropriate intervention.
